# The nonhallucinogenic ketamine metabolite (2R,6R)-hydroxynorketamine is a novel analgesic in animal models of pain

**DOI:** 10.3389/fpain.2026.1749334

**Published:** 2026-01-30

**Authors:** Bruno Carabelli, Caroline A. Browne, Irwin Lucki

**Affiliations:** 1 Department of Pharmacology & Molecular Therapeutics, Uniformed Services University of the Health Sciences, Bethesda, MD, United States; 2 Henry M. Jackson Foundation for Military Medicine, Bethesda, MD, United States; 3 Department of Biological Sciences, Munster Technological University, Cork, Ireland

**Keywords:** (2R, 6R)-hydroxynorketamine, animal models, drug therapy, glutamate, ketamine, pain

## Abstract

Current treatment options for acute and chronic pain provide limited efficacy and safety. There is an urgent need to develop drugs with new, non-opioid treatment strategies that produce fewer adverse consequences. Preclinical evidence across multiple models of acute and chronic pain demonstrate that (2R,6R)-Hydroxynorketamine [(2R,6R)-HNK], a nonhallucinogenic metabolite of ketamine, promotes potent and long-lasting analgesic effects. This review summarizes the growing evidence for the analgesic action of (2R,6R)-HNK in rodent models of acute and chronic pain. (2R,6R)-HNK produces antinociceptive effects in studies using standard tests for acute pain such as the hot plate test, although not in all studies, as well as reversal of mechanical hypersensitivity in models of acute pain like the carrageenan model (inflammatory pain). However, the most consistent anti-allodynic effects are seen in animal models aimed at mimicking chronic pain conditions, such as models of neuropathic pain (Spared Nerve Injury and Chemotherapy-induced peripheral neuropathy), low-back pain (disc puncture), complex regional pain syndrome type-1 (tibial fracture) and chronic primary pain (low-frequency percutaneous electrical nerve stimulation). Unlike ketamine, doses of (2R,6R)-HNK that counteract pain hypersensitivity do not cause sedation, dissociation, or sustain self-administration associated with abuse liability. Furthermore, distinct pharmacological effects of (2R,6R)-HNK, longer functional duration of action, non-opioid-mediated analgesia, and glutamatergic-mediated mechanisms, may distinguish (2R,6R)-HNK from ketamine and other analgesic drugs and contribute to the treatment of acute and chronic pain.

## Introduction

Ketamine has been used as an effective treatment for acute and chronic pain in both animal models (1, 2) and clinical trials (3–5), and it was recently approved by the U.S. Food and Drug Administration (FDA) for the management of surgical pain (KETARx™, PharmaTher). Interest in the use and further development of ketamine for pain indications is high due to the medical need for effective non-opioid analgesics that would reduce the reliance on opioids. However, widespread adoption of ketamine may be limited by key adverse effects that include dissociation, sedation, and abuse potential. Metabolites of ketamine are being considered as alternatives if they might share a therapeutic indication for pain without producing key side effects. Ketamine is metabolized via cytochrome P450 enzymatic transformations into several metabolites, including (2R,6R)-Hydroxynorketamine [(2R,6R)-HNK] after demethylation and hydroxylation (6). Preclinical studies have highlighted (2R,6R)-HNK as a potential alternative to ketamine for treating depression because it produces a similar pattern of behavioral effects on preclinical tests for antidepressant activity but differs from ketamine in not producing behaviors related to sedation and abuse potential (7). Recent interest in developing (2R,6R)-HNK for the treatment of acute and chronic pain conditions has also emerged from the pattern of results shown by numerous preclinical studies. Since (2R,6R)-HNK may be a safer and more effective alternative to ketamine for pain management, this minireview aims to summarize the growing evidence regarding the analgesic effects of (2R,6R)-HNK in multiple animal models of acute and chronic pain and its potential mechanisms of action.

## (2R,6R)-HNK analgesic effects in animal models

### Efficacy of (2R,6R)-HNK in models of acute pain

Acute pain conditions are defined as pain triggered by noxious stimuli, usually sudden in onset, that can arise from injury, trauma, or surgery. Acute pain evoked by a noxious stimulus can present as momentary pain or persist for days to weeks. This type of pain usually lessens or stops as healing occurs. Acute pain tests include responses to thermal sensitivity, noxious chemicals or irritants, and painful touch following an incision or paw inflammation (8).

The analgesic effects of (2R,6R)-HNK were reported using plantar incision as a model of acute postoperative pain by Kroin et al. in female CD-1 mice (9) and Das et al. in male and female CD-1 mice (10). In those reports, multiple intraperitoneal (i.p) injections of (2R,6R)-HNK (10 mg/kg daily for 3 days) counteracted the mechanical hypersensitivity measured by von Frey thresholds at 24 h after each treatment at sites ipsilateral to the injury (9, 10). The analgesia persisted for up to 5 days after the third injection. Similar results were observed in mice tested for thermal hyperalgesia using a radiant bulb heat focused on the mid-plantar surface of the left hind paw (9).

These results were confirmed and extended with dose-response curves and sex differences by Yost and colleagues (11, 12). They first showed that 10 mg/kg (2R,6R)-HNK (i.p) produced antinociception in a hot plate test in male and female C57BL/6J mice when tested 24 h after injection (11). In contrast, ketamine (10–30 mg/kg, i.p) produced only a rapid and short-lived response at 10 min after injection. No effects were observed for (2S,6S)-HNK. Subsequently, they also showed that (2R,6R)-HNK reduced mechanical and thermal hypersensitivity produced by inflammatory pain in male and female C57BL6/J mice following injection of λ-Carrageenan (CARR) in the hind paw (12). In that report, (2R,6R)-HNK reversed mechanical hypersensitivity induced by CARR at 1, 4, and 24 h with doses of 10 and 30 mg/kg (2R,6R)-HNK. The effects of (2R,6R)-HNK were comparable to carprofen, a standard non-steroidal anti-inflammatory drug (NSAID) (12).

Using an intranasal (IN) route of administration, Goswami et al. examined the antinociceptive effects of (2R,6R)-HNK in male C57BL/6J mice using the hot plate test, the Hargreaves plantar test, and persistent pain using the formalin test (13). (2R,6R)-HNK (10 mg/kg, IN) increased response latency on the hot plate at 15–60 min after treatment and in the plantar test at 30 min. Pretreatment with (2R,6R)-HNK significantly decreased spontaneous pain behavior scores (total time spent in licks and bites) in the second phase of the formalin test at 15 min and 30 min post-injection. The treatment did not alter locomotion (total distance traveled in the open field) but did increase time spent in the center of the arena and time spent grooming. No meaningful changes were found in hemodynamic or electroencephalographic parameters after (2R,6R)-HNK treatment (13).

In contrast, a study by Hillhouse et al. (2024) in male and female C57BL/6 mice (bred in-house) reported that (2R,6R)-HNK lacked efficacy on pain-stimulated and pain-depressed behavioral assays. Given 30 min before the hot plate test at 52°C, (2R,6R)-HNK (i.p) did not produce antinociceptive effects or alleviate decreased withdrawal latency at 56°C when tested after 24 h, possibly indicating hyperalgesia. Additionally, (2R,6R)-HNK failed to reverse the acetic acid-induced abdominal writhing response or the associated decrease in locomotor activity and rearing. The absence of analgesia was consistent even when (2R,6R)-HNK was administered intermittently over six days (14). These results contrast with previous findings by Yost et al., who reported that (2R,6R)-HNK increased withdrawal latency in a 50°C hot plate test (11). The discrepant results could be due to methodological differences, such as the hot plate temperatures, pretreatment time, and source of the C57BL/6 mice. Another report showed that a racemic mixture of 10 or 30 mg/kg subcutaneous (s.c.) 6-hydroxynorketamine (6-HNK) was ineffective in the tail flick, hot plate, and paw pressure tests in male Sprague-Dawley rats when tested up to 90 min after injection, whereas 10 mg/kg s.c. ketamine was effective (15). Although these testing times were appropriate in the context of ketamine, it is now apparent from more recent studies that the onset of (2R,6R)-HNK's behavioral effects occur several hours post treatment.

### Efficacy of (2R,6R)-HNK in models of chronic pain

Chronic pain is a major health problem that negatively impacts the quality of life of patients and imposes enormous socio-economic costs, with a prevalence of around 21% in the general population (16). In contrast to acute pain, chronic pain is defined as pain persisting for a period extending beyond the resolution of the primary injury. A number of key features differentiate chronic from acute pain models (17, 18), such as hypersensitivity, appearing as spontaneous pain (pain in the absence of an external stimulus), allodynia (pain resulting from an innocuous stimulus), or hyperalgesia (an exaggerated pain response to a noxious stimulus) (8).

Neuropathic pain (NP) is a common chronic pain syndrome caused by central or peripheral lesions of the nervous system that affects 7%–8% of the general population and is highly debilitating (19). Also associated with diabetes or cancer chemotherapeutic drugs, NP elicits sensory alterations, including dysesthesia and paresthesia, spontaneous pain, allodynia, and hyperalgesia (20). Animal models of NP have been developed, mainly in rodents, to recapitulate one or several symptoms of NP aimed at deciphering the underlying mechanisms. These models are distinguished according to their localization, central or peripheral, or cause of the nerve injury. Due to the limited evidence regarding the effects of (2R,6R)-HNK, here we will focus on peripheral neuropathy.

The Spared Nerve Injury (SNI) model is a classic model of NP in which the left peroneal and sural branches of the sciatic nerve are cut, and the tibial nerve is spared (21). However, variations regarding which branches are cut or preserved are used (22). Mechanical allodynia develops in the corresponding hind paw within a few days of transection and can last for months (21). Kroin et al. and Das et al. observed that treatment with (2R,6R)-HNK 10 mg/kg (i.p) increased Von Frey thresholds in SNI mice for periods up to 24 h after a single injection (female CD-1 mice were used for Kroin et al., 2019 and both male and female CD-1 mice for Das et al. In contrast, ketamine 10 mg/kg (i.p) was ineffective (9). Furthermore, 3 daily injections of (2R,6R)-HNK 10 mg/kg spaced 24 h apart reduced allodynia produced by SNI (9, 10). Similar results were found by Yost et al, measuring mechanical hypersensitivity with the Von Frey test in the SNI model in male and female C57BL/6J mice. A single injection of 10 or 30 mg/kg (2R,6R)-HNK increased paw withdrawal thresholds at 4 h and 24 h after treatment. In addition, the effects of (2R,6R)-HNK were equivalent in magnitude but longer lasting than gabapentin (10 or 30 mg/kg, i.p), a medication frequently prescribed for NP (11). A clinical trial evaluating the effects of (2R,6R)-HNK in chronic neuropathic pain is currently underway (NCT05864053).

Another model of chronic pain is the Tibial Fracture/Complex regional pain syndrome type-1 (CRPS1). Injection of (2R,6R)-HNK for 3 days produced an antiallodynic effect in CRPS1 pain measured at 24 h after each injection, and the analgesia lasted 4 days beyond treatment cessation using both male and female CD-1 mice (9, 10). The behavioral effect of (2R,6R)-HNK persisted beyond the period expected from pharmacokinetic exposure since the half-life of (2R,6R)-HNK in the mouse brain is less than 1 h (6, 23). This suggests that (2R,6R)-HNK may cause a sustained reduction of the central sensitization processes that contribute to lasting CRPS1 pain (24).

Using female C57BL/6 mice, Liu et al. demonstrated that intrathecal administration of (2R,6R)-HNK (7–21 uM), rather than intraperitoneal (2R,6R)-HNK (10 mg/kg) or intrathecal S-ketamine (7–21 uM), successfully mitigated pain induced by acute or repeated High-Frequency Electrical Stimulation (HFS). Using a low-frequency percutaneous electrical nerve stimulation (LF-PENS) to directly stimulate peripheral pain pathways, intrathecal (2R,6R)-HNK was effective in attenuating bilateral pain hypersensitivity. In addition, (2R,6R)-HNK reduced cognitive and depressive-like behaviors in a dose-dependent manner (25).

In a recent study, Das et al. investigated the analgesic effects of (2R,6R)-HNK in a murine model of lumbar disk puncture, both alone and in combination with NSAID meloxicam (26). The tactile allodynia induced by their model was reduced dose-dependently by (2R,6R)-HNK. The estimated effective dose 50% (EC50) for (2R,6R)-HNK was 14.2 mg/kg in male mice and 16.9 mg/kg in female mice, indicating no significant sex difference in its efficacy. Meloxicam and (2R,6R)-HNK co-administration enhanced the analgesic effect on paw withdrawal compared to meloxicam alone.

Recently, our group has shown that (2R,6R)-HNK also reversed thermal and mechanical allodynia in a model of chemotherapy-induced peripheral neuropathy (CIPN) produced by oxaliplatin treatment in rats. There was no indication of the development of tolerance to the analgesia following repeated treatment for 7 days. Moreover, the analgesia persisted for about 2 weeks after cessation of treatment. In addition, this study is the first demonstration of analgesic effects in rats (27).

## Mechanisms underlying (2R,6R)-HNK analgesia

### Pharmacological mechanisms of action

Several studies have explored the pharmacological mechanisms associated with the effects of (2R,6R)-HNK in murine models of acute and chronic pain. A common finding is that the antiallodynic effects of (2R,6R)-HNK were not blocked by the opioid receptor antagonists naloxone or naltrexone, indicating that the effects of (2R,6R)-HNK in models of acute and chronic pain do not involve the activation of opioid receptors (9–11). In contrast, the antinociceptive effects of ketamine have been shown to be blocked by pretreatment with opioid antagonists (11, 28), suggesting important differences in the mechanisms between ketamine and (2R,6R)-HNK associated with analgesia.

Glutamatergic α-amino-3-hydroxy-5-methyl-4-isoxazolepropionic acid (AMPA) receptors, on the other hand, are involved in mediating the analgesic effects of (2R,6R)-HNK. Using antinociceptive behavior on the hot plate test or the CARR model of inflammatory pain, pretreatment with the AMPA receptor antagonist NBQX prevented both the development and expression of (2R,6R)-HNK-induced analgesia, but not the analgesic effects of ketamine (11, 12). Similarly, Das et al. reported that analgesic effects on the plantar incision, tibial fracture, and SNI models by (2R,6R)-HNK were prevented by pretreatment with NBQX. In addition, in the hippocampus of mice exposed to the SNI and CRPS1 pain models, (2R,6R)-HNK was shown to reverse deficits in AMPA protein subunits GluA1 and GluA2, enhanced levels of phosphorylated Calcium/Calmodulin-Dependent Protein Kinase II, (p-CaMKII), which phosphorylates GluA1, and reversed changes in expression of both voltage activated potassium channel, p-Kv2.1 (elevated expression levels), and Brain Derived Neurotrophic Factor (BDNF) (decreased expression levels), which are critical for maintenance of intrinsic excitability and plasticity (10).

Using pharmacological experiments, Liu et al. observed that the Transient Receptors Potential Ankyrin 1 (TRPA1) inhibition was the main mechanism by which intrathecal (2R,6R)-HNK exerts its analgesic effects in the LF-PENS model. Moreover, they showed that (2R,6R)-HNK attenuated neuronal hyperexcitability and upregulation of calcitonin gene-related peptide (CGRP), TRPA1, and vesicular glutamate transporter-2 (VGLUT2) in the peripheral nociceptive pathway. Additionally, (2R,6R)-HNK suppressed calcium responses and CGRP overexpression in cultured DRG neurons (25).

Das et al. showed that (2R,6R)-HNK analgesia in a lower back pain model was associated with significant protein changes across different nervous system regions. Similar to the group's previous work in the hippocampus, (2R,6R)-HNK treatment led to increased amounts of GluA1, GluA2, p-Kv2.1, and p-CaMKII. Concurrently, there was a reduction in BDNF protein ratios in both the hippocampus and the dorsal root ganglion (DRG). The study also observed that (2R,6R)-HNK attenuated the upregulation of c-Fos in the spinal cord, a marker often associated with neuronal activity in pain pathways. Additionally, novel findings included a decrease in CXCR4 and p-AKT protein ratios in the hippocampus. These protein analyses collectively suggest that (2R,6R)-HNK's analgesic activity involves modulating central and potentially peripheral pain processes, supporting its continued investigation as a non-opioid analgesic for discogenic back pain. Furthermore, the antiallodynic effect of (2R,6R)-HNK was blocked by pretreatment with NBQX, but notably not by naloxone, again suggesting a non-opioid mechanism of action (26).

Overall comparisons between the analgesic effects of (2R,6R)-HNK and ketamine suggest that they produce their effects by different mechanisms. Ketamine analgesia is produced rapidly after administration but dissipates within hours. (2R,6R)-HNK has been reported to produce rapid effects within hours after injection, but its analgesic effects typically last for 24 h or longer after a single administration. After cessation of repeated treatment, several studies reported persistent analgesic effects lasting for days to weeks (9, 10, 27). (2R,6R)-HNK remains detectable in plasma and the brain up to 4 h after a single dose in mice (6). The reasons for a longer duration of analgesic effects than expected from drug exposure remain to be explained but are consistent with suggestions that (2R,6R)-HNK produces its effects by causing long-lasting changes in neuroplasticity, particularly in the presence of chronic pain (29).

Overall, despite the growing evidence of its analgesic properties, the primary molecular target of (2R,6R)-HNK remains unknown. Target deconvolution studies have thus far failed to identify a direct binding site (30). Consequently, the current mechanistic understanding relies largely on downstream effects observed via pharmacological antagonism and protein analysis.

## Summary and future directions

Despite a pressing medical need, translating drug effects on pain from animals to humans has been challenging. Biological differences in pain biology, perception, and social factors unique to medical evaluation in humans contribute to a low success rate among candidates (31). This review summarized the cumulative evidence for the antinociceptive or analgesic effects of (2R,6R)-HNK from 9 published studies using 10 models of acute or chronic pain in mice, and 1 study in rats (Table 1). While several studies using acute pain models report efficacy, overall findings remain variable. As mentioned previously, Hillhouse et al. reported a lack of efficacy in pain-stimulated and pain-depressed behaviors, including the hot plate and acetic acid writhing tests (14). This contrasts with Yost et al., who found significant antinociception in the hot plate test (11). These discrepancies may stem from methodological variations, such as the specific hot plate temperature (52°C vs. 50°C) or substrain differences in C57BL/6 mice, which are known to influence nociceptive sensitivity. Additionally, Lilius et al. found that a racemic mixture of 6-HNK was ineffective in rat models of acute thermal and mechanical pain (15), although longer testing times may be more effective. That the efficacy of (2R,6R)-HNK in acute nociception tests is variable and modest is not unexpected and consistent with calls for the greater use of chronic pain models with enhanced medical validity in analgesic drug discovery (31).

**Table 1:** Effects of (2R,6R)-HNK in murine models of acute and chronic pain.

Pain model	Pain type	Primary behavioral outcome	Dose & route	Species/strain/sex	Time of effect/key finding	Ref
Plantar incision	Acute	Mechanical Hypersensitivity (von Frey)	10 mg/kg (IP); Single & Daily ×3	Mice/CD1/M; F.	**Sustained**: Analgesia persisted 24 h after injection and up to 5 days after repeated dosing.	(9, 10)
Hot plate	Acute	Thermal Nociception	10 mg/kg (IP); Single	Mice/C57BL/6J/M; F.	**Delayed**: antinociception observed at 24 h post-injection	(11)
λ-Carrageenan	Acute	Mechanical & Thermal Hypersensitivity	10–30 mg/kg (IP); Single	Mice/C57BL/6J/M; F.	**Rapid & Sustained**: Reversal of hypersensitivity at 1 h, 4 h and 24 h.	(12)
Hargreaves/hot plate/formalin test	Acute	Thermal/Spontaneous Pain	10 mg/kg (IN); Single	Mice/C57BL/6J/M.	**Rapid**: Analgesia observed 15–60 min post-administration; decreased spontaneous pain (Phase II)	(13)
Hot plate/writhing	Acute (Stimulated)	Thermal Nociception/Abdominal Writhing	10 mg/kg + (IP); Cumulative	Mice/C57BL/6N/M; F.	**No Effect**: failed to produce antinociception at 30 min or 24 h.	(14)
SNI	Chronic	Mechanical Hypersensitivity (von Frey)	10–30 mg/kg (IP); Single & Daily ×3	Mice/CD1/C57BL/6J/M; F.	**Sustained**: Increased thresholds at 4 h and 24 h.	(9–11)
CRPS-1	Chronic	Mechanical Hypersensitivity (von Frey)	10 mg/kg (IP); Daily ×3	Mice/CD1/C57BL/6J/M; F.	**Sustained**: Analgesia persisted 24 h after each dose and 4 days post-cessation.	(9, 10)
LF-PENS	Chronic	Mechanical Hypersensitivity (von Frey)	10 mg/kg (IT, IP); Single/Multi	Mice/C57BL/6/F.	**Variable**: Attenuated bilateral hypersensitivity; IT route effective.	(25)
Lumbar disk puncture	Chronic	Mechanical Hypersensitivity (von Frey)	∼14–17 mg/kg (IP); Daily ×3	Mice/C57BL/6J/M; F.	**Sustained**: Dose-dependent reduction in allodynia observed 24 h after each injection.	(26)
CIPN	Chronic	Mechanical & Thermal Allodynia	30 mg/kg (SC); Single & Daily ×7	Rats/Sprague-Dawley/M; F.	**Long-lasting**: Analgesia persisted for 2 weeks following cessation of 7-day treatment.	(27)

SNI, spared nerve injury; LF-PENS, low-frequency percutaneous electrical nerve stimulation; IP, intraperitoneal; iN, intranasal; IT, intrathecal; CIPN, chemotherapy-induced peripheral neuropathy; M, male; F, female.

In fact, consistent demonstrations of analgesic effects for (2R,6R)-HNK were obtained across multiple models involving persistent pain intended to translate conditions with major unmet medical needs. These include neuropathic pain induced by nerve injury or cancer chemotherapeutic agents, CRPS, lower back pain, and chronic high-frequency electrical stimulation. Significant effects of (2R,6R)-HNK were reported with both male and female mice using either the CD-1 outbred or C57BL/6 inbred strain. One positive report was obtained with rats. The most commonly used dose was 10 mg/kg, and most studies used the intraperitoneal injection route, but the intranasal, intrathecal, or subcutaneous routes were also used in separate studies. Taken together, these early preclinical investigations of (2R,6R)-HNK justify continued research to determine its precise mechanism of action and whether they translate into effective and safe management strategies for pain patients.

The analgesic effects of (2R,6R)-HNK may contribute to those of ketamine, acting differently from ketamine itself, when used to treat some chronic pain conditions, such as CRPS. Nevertheless, (2R,6R)-HNK may provide a critical advance in safety and efficacy because some adverse effects of ketamine in humans, such as dissociation, sedation, and abuse potential, are not produced by (2R,6R)-HNK in corresponding mouse models (23, 30). A Phase 1 trial conducted with normal volunteers confirmed that (2R,6R)-HNK did not produce significant sedation, dissociative effects, and a minimal number of adverse events at the doses examined (32). Clinical trials for (2R,6R)-HNK in neuropathic pain (NCT05864053), depression (NCT06511908), and obsessive-compulsive disorder (NCT06575075) are currently underway. Evaluation of the relative safety of (2R,6R)-HNK will remain one of the key points driving its future development for depression, pain, and other indications.

In addition to the preclinical indications for efficacy against pain, pharmacological findings have distinguished (2R,6R)-HNK from other analgesic drugs. For example, (2R,6R)-HNK produced a long-lasting analgesic response from a single dose that lasted for 24 h or longer. In addition, (2R,6R)-HNK also produced analgesic effects that persisted for days to weeks following the cessation of repeated treatment, although the mechanism of these lasting effects is unknown. The time of exposure to (2R,6R)-HNK in the mouse is relatively short, with the half-life in brain and plasma under 1 h and exposure for 4 h (6), so that sustained exposure to (2R,6R)-HNK levels would be unlikely to account for these persistent effects on pain. Important questions for studies of pain models to address are whether a similar temporal pattern of effects can be expected with other species and what the mechanism is. If confirmed in humans, this feature could make (2R,6R)-HNK especially useful for many chronic pain disorders.

Another issue affecting the clinical development of any medication for pain is whether the effects of the drug are maintained following chronic treatment due to the development of tolerance or adverse effects. Thus far, (2R,6R)-HNK has not shown tolerance to its analgesic effects in rats (27). Although (2R,6R)-HNK has been compared with other medications in different pain models, no studies have comprehensively compared the effects of (2R,6R)-HNK with other medications across a range of doses and treatment conditions. Studies also need to evaluate the interaction of (2R,6R)-HNK with other established analgesics, such as NSAIDs and opioids, to determine its role in providing adjunctive pain treatment.

Some information about the mechanisms of action of (2R,6R)-HNK relevant to analgesia has been derived from the use of pharmacological antagonists (detailed in the previous section). However, its precise molecular targets are still unknown, and no direct primary target for (2R,6R)-HNK has been reported (30). Glutamatergic systems appear to be the most promising mechanism for (2R,6R)-HNK and have been proposed to be involved in regulating acute and chronic pain (33). Translational studies with informative biomarkers will be crucial to confirm the efficacy and safety of (2R,6R)-HNK on pain conditions and elucidate its physiological mechanisms of action (34).

It is important to highlight that significant translational gaps remain between murine models and human clinical applications. First, the pharmacokinetic profile in rodents, characterized by a rapid half-life, contrasts with the sustained analgesia observed, raising questions about whether this temporal dissociation will replicate in humans. Second, while phase 1 trials confirm safety, head-to-head comparisons with standard-of-care analgesics (e.g., NSAIDs, gabapentinoids) are limited in the preclinical literature. Finally, the variability of efficacy in acute pain models suggests that (2R,6R)-HNK may be more suited for central sensitization phenotypes (chronic pain) rather than acute nociception, a distinction that must guide clinical trial design.
